# Efficient Modulation of Exon Skipping via Antisense Circular RNAs

**DOI:** 10.34133/research.0045

**Published:** 2023-01-19

**Authors:** Shuaiwei Ren, Mei Huang, Raoxian Bai, Lijiao Chen, Jiao Yang, Junyu Zhang, Wenting Guo, Weizhi Ji, Yongchang Chen

**Affiliations:** ^1^ State Key Laboratory of Primate Biomedical Research; Institute of Primate Translational Medicine, Kunming University of Science and Technology, Kunming 650500, China.; ^2^ Yunnan Key Laboratory of Primate Biomedical Research, Kunming 650500, China.

## Abstract

Splice-switching antisense oligonucleotides (ASOs) and engineered U7 small nuclear ribonucleoprotein (U7 Sm OPT) are the most commonly used methods for exon skipping. However, challenges remain, such as limited organ delivery and repeated dosing for ASOs and unknown risks of by-products produced by U7 Sm OPT. Here, we showed that antisense circular RNAs (AS-circRNAs) can effectively mediate exon skipping in both minigene and endogenous transcripts. We also showed a relatively higher exon skipping efficiency at the tested *Dmd* minigene than U7 Sm OPT. AS-circRNA specifically targets the precursor mRNA splicing without off-target effects. Moreover, AS-circRNAs with adeno-associated virus (AAV) delivery corrected the open reading frame and restored the dystrophin expression in a mouse model of Duchenne muscular dystrophy. In conclusion, we develop an alternative method for regulating RNA splicing, which might be served as a novel tool for genetic disease treatment.

## Introduction

Exon skipping is a promising therapeutic strategy, which typically removes the affected exon to produce truncated proteins with partial or full function for alleviating disease phenotypes. Although splice-switching antisense oligonucleotides (ASOs) and engineered U7 small nuclear ribonucleoprotein (U7 Sm OPT) are the most commonly used tools, they both have limitations. ASO could not be delivered by adeno-associated virus (AAV) and required repeated administrations [1]. U7 Sm OPT may produce by-products with unknown risks [2]. Thus, more alternative tools need to be developed.

Circular RNA (circRNA) is a class of closed-loop single-stranded RNAs [3]. They are more stable than their cognate linear RNAs and show great application potential in biomedicine [4], such as mRNA vaccines. Here, we develop a new alternative approach that uses antisense circRNA (AS-circRNA) for exon skipping. We show that cyclization could increase the stability of antisense RNAs (AS-RNAs) and effectively mediate exon skipping both in vitro and in vivo, indicating that AS-circRNA could be served as a potential therapeutic strategy.

## Results

### AS-circRNAs effectively mediate exon skipping in a *Dmd* minigene and endogenous transcripts

**Fig. 1.**
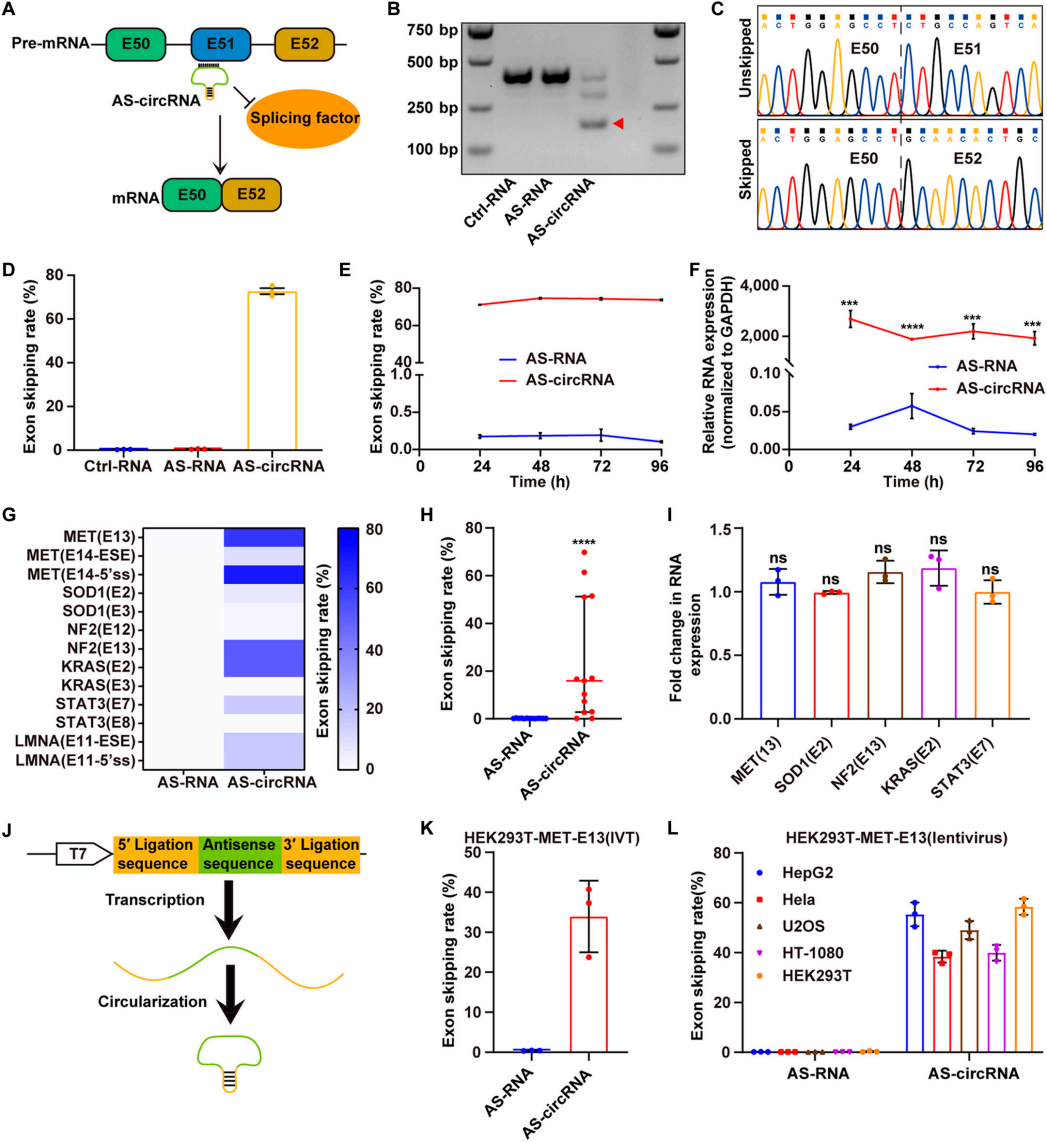
AS-circRNAs effectively mediate exon skipping in a *DMD* minigene and endogenous transcripts. (A) Schematic diagram of the *DMD* minigene and the working principle behind AS-circRNA. The minigene is composed of exons 50, 51, and 52 of the mouse *DMD* gene. The AS-circRNA binds to exon 51 of the precursor mRNA and impedes the binding of splicing factors, resulting in exon 51 skipping. (B) Detection of exon 51-skipped mRNA by RT-PCR in the AS-circRNA but not AS-RNA (linear) or Ctrl-RNA (random antisense sequence) groups. (C) Sanger sequencing of RT-PCR amplified products in the AS-circRNA group. (D) Quantitative analysis of the exon skipping rate in the different groups; *n* = 3, mean ± SD. (E) Quantitative analysis of the exon skipping rate at different time points after transfection of HEK293T cells with either an AS-circRNA or an AS-RNA plasmid; *n* = 3, mean ± SD. (F) The relative expression levels of AS-circRNAs and AS-RNAs at different time points after transfection of HEK293T cells, normalized to GAPDH. Significance was determined by 2-tailed unpaired Student’s *t* test; *n* = 3, mean ± SD. (G) Heat map showing the exon skipping rate mediated by different AS-circRNAs and AS-RNAs, targeting endogenous transcripts including MET, SOD1, KRAS, NF2, STAT3, and LMNA in HEK293T cells. (H) The exon skipping rates induced by AS-circRNA and AS-RNA. Mann–Whitney U test was used for significance analysis. Midline: the median; limits: 75% and 25%. (I) Fold change of target transcript expression normalized to the Ctrl-RNA group 48 h after transfection; *n* = 3, mean ± SD. Significance was determined by 2-tailed unpaired Student’s *t*-test; *n* = 3, mean ± SD. (J) Schematic diagram of an AS-circRNA synthesized by in vitro transcription and cyclization. (K) The exon skipping rate of the *MET* gene targeted by in vitro-synthesized AS-circRNA in HEK293T cells, 24 h after transfection; *n* = 3, mean ± SD. (L) The exon skipping rate of the *MET* gene in different cell lines transfected by lentivirus, 96 h after transfections; *n* = 3, mean ± SD. ***P < 0.001 and ****P < 0.0001; ns, *P* ≥ 0.05.

To assess if the AS-circRNA could mediate exon skipping, we first constructed a minigene, *Dmd* (exon 50–52), the causing gene of Duchenne muscular dystrophy (DMD). DMD is an X-linked recessive disorder that causes skeletal muscle weakening and eventual death by heart and respiratory failure. ASO-mediated exon skipping has been used to treat the disorder [5]. We designed and cyclized an antisense sequence that targets the exon 51 of the *Dmd* minigene (Dmd^Exon51^-AS-circRNA) using genetically encoded methods (Fig. 1A and Fig. S1A) [6]. The cyclization of antisense sequence was confirmed by reverse transcription polymerase chain reaction (RT-PCR) and Sanger sequencing (Fig. S1B and C). After transfecting the *Dmd* minigene and AS-circRNA plasmids into human embryonic kidney 293T (HEK293T) cells, we observed skipped bands in the AS-circRNA group, but not in the AS-RNA group (linear AS-RNA) or Ctrl-RNA group (cyclizing random antisense sequence) (Fig. 1B). Sanger sequencing also confirmed the complete skipping of exon 51 (Fig. 1C). The exon skipping rate was about 72% in the AS-circRNA group and less than 0.5% in other groups (Fig. 1D). We found that the exon skipping rate peaked at 48 h in the AS-circRNA group and remained stable at 72 and 96 h, whereas the linear RNA group did not show a significant change (Fig. 1E). Consistent with these findings, the quantitative PCR (qPCR) data showed that the circRNAs maintained a dramatically higher level compared to linear RNA at the time points being tested (Fig. 1F). These results indicate that AS-circRNAs are stable and can effectively mediate exon skipping in a sequence-dependent manner. We further showed that circularization is required for exon skipping and RNA stability by using defective ribozymes (Fig. S2) [7]. Once certain parameters are met (e.g., sufficient concentration and length and optimal targeting sequence), AS-circRNAs can efficiently promote exon skipping in *Dmd* minigene (Fig. S3). Moreover, compared to U7 Sm OPT-5' splice site (U7 Sm OPT-5'ss) and U7 Sm OPT-exonic splicing enhancer (U7 Sm OPT-ESE), both AS-circRNA-5’ss and AS-circRNA-ESE showed a higher skipping rate (Fig. S3D).

We next explored whether AS-circRNAs could also mediate the exon skipping of endogenous transcripts in HEK293T cells. We constructed 13 AS-circRNA plasmids targeting 11 exons of 6 endogenous transcripts (MET, KRAS, SOD1, STAT3, NF2, and LMNA). Forty-eight hours after transfection, 9 of 13 AS-circRNAs could mediate exon skipping, with the highest skipping rate observed for the MET transcript (E14-DS; more than 70%). In contrast, none of AS-RNAs could mediate exon skipping (Fig. 1G and H and Fig. S4). Notably, we did not observe a significant change in RNA expression of targeted endogenous transcripts (Fig. 1I). We also tested whether in vitro-synthesized AS-circRNAs could promote exon skipping (Fig. 1J). We used the MET gene as an example and found that in vitro-synthesized AS-circRNAs through liposome delivery could mediate an effective MET exon 13 skipping in HEK293T cells (Fig. 1K). We also compared in vitro-synthesized AS-circRNAs with the ASO (Eteplisren), which is one of the Food and Drug Administration-approved ASOs for the DMD treatment (Fig. S5). The in vitro*-*synthesized AS-circRNA and ASO (Eteplisren) showed comparable efficiency in mediating the exon skipping of minigene transcripts, but ASO (Eteplisren) showed more long-lasting effects. Through lentivirus infection, the AS-circRNA that targets MET exon 13 could play functions in a range of cell lines, including HEK293T, HeLa, HepG2, U2OS, and HT-1080, indicating that AS-circRNAs have broad applicability to a variety of cell types (Fig. 1L). No detectable off-target effects were observed (Fig. S6 and Table S1). Collectively, our data demonstrate that AS-circRNAs could mediate exon skipping in endogenously expressed genes.

### AS-circRNAs restore dystrophin protein expression in DMD mouse model

**Fig. 2.**
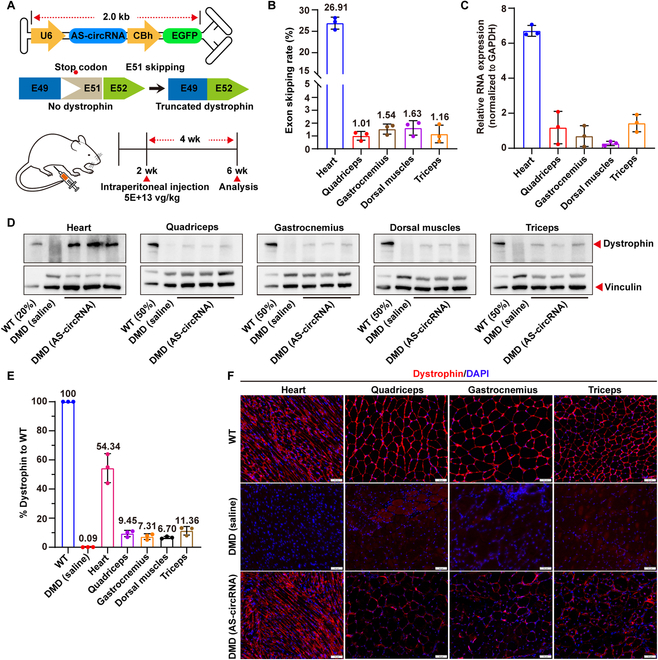
AS-circRNAs restore dystrophin protein expression in DMD mouse model. (A) Schematic diagram depicting the in vivo experimental design. The AS-circRNA, previously tested in vitro for DMD E51 skipping, was packaged into scAAV. AAV was injected intraperitoneally into 2-week-old DMD mice, and the mice were euthanized for analysis 4 weeks after injection. Different muscles were assessed to determine whether the correct DMD reading frame and dystrophin protein expression were restored. (B) Quantification of exon 51 skipping rate in DMD mouse muscle tissues including heart, quadriceps, gastrocnemius, dorsal muscles, and triceps muscles 4 weeks after AAV injection. *n* = 3, mean ± SD. (C) Relative expression of AS-circRNA in the heart, quadriceps femoris, gastrocnemius, dorsal muscle, and brachial muscle of AAV-injected mice, normalized to GAPDH; *n* = 3, mean ± SD. (D) Western blot analysis showing that dystrophin expression was restored in the heart, quadriceps, gastrocnemius, dorsal muscles, and triceps muscles of DMD mice 4 weeks after systemic injection. (E) Percentage of recovered dystrophin protein in the gastrocnemius, quadriceps, triceps, dorsal muscles, and heart. Dystrophin percentage was first normalized to vinculin and compared to WT. Images were analyzed by ImageJ. *n* = 3, mean ± SD. (F) Immunofluorescence staining of dystrophin in the gastrocnemius, quadriceps, triceps, and heart muscle. Scale bars, 50 μm.

Given the availability and specificity of AS-circRNAs in vitro, we investigated whether AS-circRNAs could be employed as a therapeutic tool in vivo. We constructed a mouse model lacking exon 50 of the *Dmd* gene using the CRISPR/Cas9 system [8]. To restore the mutated reading frame, we packaged the previously tested Dmd^Exon51^-AS-circRNA and enhanced green fluorescent protein (EGFP) into the self-complementary MyoAAV vector (Fig. 2A). MyoAAV was injected intraperitoneally into 2-week-old mice. Four weeks later, we found that EGFP mainly distribute in muscle tissues and kidneys and a little in the lungs and liver (Fig. S7A), which is consistent with the previous reports [9]. Exon skipping efficiency was highest in the heart (26.91%), 1.01% in the quadriceps, 1.54% in the gastrocnemius, 1.63% in the dorsal muscles, and 1.16% in the triceps muscles (Fig. 2B and Fig. S7B). In addition to the exon-skipped products, we also observed unintended bands, which was due to the alternative 3' splice site (3'ss) selection (Fig. S7B to D). The heart has the highest expression of AS-circRNAs (Fig. 2C), which may explain the highest skipping rate in the heart. Furthermore, DMD mice injected with MyoAAV-AS-circRNA showed widespread expression of dystrophin protein in the heart, quadriceps, gastrocnemius, dorsal muscles, and triceps muscles (Fig. 2D to F). Expression of the AS-circRNA restored dystrophin protein expression to 54.34% in the heart, 9.45% in the quadriceps, 7.31% in the gastrocnemius, 6.70% in the dorsal muscles, and 11.36 % in triceps muscles (Fig. 2E). AS-circRNA-treated mice showed significant improvement in both pathologic and behavioral phenotypes (Fig. S8). Together, these data demonstrated that AS-circRNA could serve as a potential therapeutic tool in DMD.

In this study, we present the first proof of concept that AS-circRNAs can mediate exon skipping both in vitro and in vivo*.* With AAV delivery, AS-circRNA can achieve broad tissue distribution. In conclusion, AS-circRNAs represent a promising alternative for splicing regulation with potential therapeutic applications.

## Materials and Methods

### Plasmid construction

To construct the *Dmd* minigene, exons 50, 51, and 52 of the mouse *Dmd* gene were amplified by PCR. The PCR products were purified using the EasyPure Quick Gel Extraction Kit (Transgen #EG101-01) and constructed into the pcDNA 3.1 vector through HindIII (New England Biolabs (NEB) #R3104) and EcoRI (NEB #R3101) restriction site using the ClonExpress II One Step Cloning Kit (Vazyme #C112). To construct a vector expressing linear AS-RNA, the PGL3 vector (Addgene #107721) was linearized by BsaI (NEB #R3733) and EcoRI (NEB #R3101). Antisense sequences of less than 50 base pairs (bp) were chemically synthesized, annealed, and ligated to the linearized PGL3 vector by T4 DNA ligase (Vazyme #C301). Antisense sequences greater than 100 bp were amplified by PCR and ligated to the linearized PGL3 vector using the ClonExpress II One Step Cloning Kit (Vazyme #C112). The transcription of AS-RNA was driven by the U6 promoter. DNA, including a Twister P3 U2A, a 5′ ligation sequence, a 3′ ligation sequence, and a Twister P1, was synthesized and cloned into a linearized PGL3 vector to construct the AS-circRNA expression vector. Antisense sequences of different lengths were ligated between the 2 ligation sequences by the method described above. The sequence of U7 has been published previously [10]. Primers and antisense sequences are listed in Table S2.

### Cell culture and transfection

Hela, HepG2, HT-1080, HEK293T, and U2OS cell lines were purchased from the Cell Bank of the Chinese Academy of Sciences. All cell lines were cultured with Dulbecco's modified Eagle's medium (DMEM) (Thermo Fisher #11965092) supplemented with 10% fetal bovine serum (BI #04-001-1) and 1% penicillin–streptomycin (Thermo Fisher #10378016) at 5% CO_2_, 37 °C.

HEK293T cells were seeded in 12-well plates (1.5 × 10^5^ cells per well) until 40 to 50% confluency. *Dmd* minigene (0.5 μg) and 1 μg of AS-circRNA plasmid were co-transfected into the HEK293T cells with Lipofectamine 3000 (Thermo Fisher #L3000150). The same amount of AS-RNA and Ctrl-RNA were used as control. Cells were obtained for subsequent experiments 48 h after transfection. To test the exon skipping of endogenous transcripts mediated by AS-circRNA, 1.5 μg of AS-circRNA plasmid was transfected into cells with Lipofectamine 3000. Cells were obtained for subsequent experiments 48 h after transfection. AS-circRNA (1 μg) synthesized in vitro was transfected into HEK293T cells for 24 h using Lipofectamine 2000 (Thermo Fisher #11668019). To assess the exon skipping rate in multiple cell lines, lentivirus was produced by OBiO Technology Corp. Ltd. (Shanghai, China) and infected HEK293T, Hela, HepG2, or HT-1080 cell lines at 10 MOI (multiplicities of infection). Cells were collected 96 h after infection. To analysis the half-life of different versions of RNA, 5 mg/ml of actinomycin D was added to the culture medium 24 h after transfection. Cells were then collected at 1, 7, 13, 19, and 25 h after actinomycin D addition for analysis.

### Animals

All the animal care and experimental procedures were approved by the Laboratory Animal Center of Kunming University of Science and Technology (permit no. PZWH (Yunnan) K2022-0023) and followed the National Institutes of Health care guidelines. Mice were housed in a specific pathogen-free (SPF) facility under normal 12-h light/12-h dark cycles. The temperature was maintained at 18 to 23 °C and humidity at 40 to 70%.

### MyoAAV injection

*Dmd* mice were generated via dual small guide RNAs (sgRNAs) based on previously reported methods [8]. MyoAAV was packaged by PackGene Biotech (Guangzhou, China). Two-week-old *Dmd* male mice were injected intraperitoneally with 100 μl of the virus at a dose of 5 × 10^13^ vg/kg. Control mice (2-week-old C57BL/6J male mice and *Dmd* male mice) were injected with an equal volume of saline. At least 3 mice were injected for each experimental condition. Mice were monitored 4 times per week for 4 weeks. The sgRNAs are listed in Table S2.

### In vitro transcription and production of AS-circRNAs

The antisense sequence for cyclization was amplified from the plasmid by PCR. The T7 promoter was added to the 5′ end of the product. The PCR products were purified with the EasyPure Quick Gel Extraction Kit (TransGen, no. EG101-01) and used for in vitro transcription. AS-circRNA precursors were synthesized by in vitro transcription using the HiScribe T7 High-Yield RNA Synthesis kit (NEB #E2040). The transcript products were further cyclized as previously reported [7]. ASO (Eteplisren) was synthesized by the company (Tsingke Biotechnology Co. Ltd.)

### RNA analysis

Cells or tissues were treated with TRIzol reagent (Life Technologies) to extract total mRNA according to the manufacturer’s protocol. One microgram of RNA was reverse transcribed to cDNA using the PrimeScript RT Reagent Kit with gDNA Eraser (Takara #RR047). The TransStart Green qPCR SuperMix Kit (Transgen #AQ101-01) was used to quantify the relative expression of genes with GAPDH as an internal reference. To quantify the exon skipping rate of endogenous transcripts, the gray scale of the PCR amplicon was calculated by ImageJ. The absolute quantification of the exon 51 skipping rate in the minigene was quantified by qPCR. Exon 50–51 or exon 50–52 was amplified using the Bio-Rad CFX384 Touch real-time PCR detection system. Plasmids containing the exon 50–51 or exon 50–52 cDNA fragment were used as standards. Exon skipping rate = (exon 50–52 copies − exon 50–51 copies)/exon 50–52 copies. The same quantification method was also applied for the exon 51 skipping rate in the DMD mouse model. The primers were designed to keep the amplicon length within 300 bp. Two replicates were performed for each sample. The primers used are listed in Table S2.

### Western blotting

Mouse tissues were lysed using the radioimmunoprecipitation assay lysis buffer (Beyotime #P0013C) supplemented with 100 μm of protease inhibitor phenylmethylsulfonyl fluoride (Beyotime #ST506) for 1 h at 4 °C. Lysates were obtained by centrifuging at 10,000×g in a microcentrifuge at 4 °C for 20 min to remove unsolubilized materials. Proteins were subjected to sodium dodecyl sulfate polyacrylamide gel electrophoresis (SDS-PAGE) gel and transferred onto Immobilon-P transfer membranes (Millipore #IPVH000010). The membranes containing the transferred proteins were blocked for 2 h with 5% skim milk at room temperature. The blocked membrane was incubated with dystrophin (Abcam #ab154277), EGFP (Abcam #ab184601), or vinculin (Cell Signaling Technology (CST) #4650S) overnight at 4 °C and then incubated with anti-rabbit immunoglobulin G (IgG) (CST #7074S) or anti-mouse IgG (CST# 7076S) secondary antibodies conjugated to horseradish peroxidase. After washing for 10 min 3 times, the bound antibodies were detected using an Immobilon ECL Ultra Western horseradish peroxidase substrate (S0500, Millipore). The experiment was repeated 3 times. The intensity of each band was analyzed by ImageJ software.

### Immunofluorescence staining

Skeletal muscles (quadriceps, gastrocnemius, and triceps) and heart were cryo-sectioned at 8 μm and fixed with 4% paraformaldehyde for 30 min. After washing with phosphate-buffered saline 3 times, 10 min each, sections were permeabilized with 0.2% Triton X-100 for 2 h and then blocked with blocking buffer containing 3% bovine serum albumin and 10% fetal bovine serum for 2 h at room temperature. Sections were then incubated with primary antibody (dystrophin, Abcam #ab15277; 1:400 dilution) overnight at 4 °C, followed by donkey anti-rabbit IgG H&L secondary antibody (Alexa Fluor 568, Abcam #ab175694) for 1 h at room temperature. Cell nuclei were stained with 4′,6-diamidino-2-phenylindole (Invitrogen; 1:1,000 dilution).

### Transcriptome sequencing analysis

Total RNA was extracted from HEK293T cells that were transfected with AS-circRNA, Ctrl-RNA, or transfection reagents (blank). mRNA was enriched using Oligo (dT) magnetic beads to construct cDNA libraries. After library construction, Qubit 3.0 was used for preliminary quantification. Agilent 2100 was used to detect the insert size of the library to ensure the quality of the library. Sequencing was performed on the NovaSeq 6000 S4 platform (NovaSeq 6000 S4 Reagent Kit V1.5). The double-ended sequencing program was run to obtain double-ended sequencing reads of 150 bp. Sequencing data were matched to human genome data (GRCm38) using hisAT2 (version 2.2.1). Potential off-target sites were identified by comparing the AS-circRNA group with the other 2 groups using DEXseq. The binding ability of AS-circRNA to the potential off-target sequences was analyzed using Miranda. The potential off-target sites are listed in Table S1.

### Histological analysis

Skeletal muscles (quadriceps, gastrocnemius, and triceps) and heart of wild-type (WT), DMD (saline), and DMD (MyoAAV) mice were cryo-sectioned at 8 μm. Hematoxylin–eosin staining (Solarbio #G1120) and Sirius red staining (Solarbio #G1472) were conducted according to manufacturer’s protocols.

### Behavioral phenotype analysis

Grip strength was measured using a mouse grip strength meter (Shanghai Merisai Scientific Instrument Co. Ltd. # MC-RMG01). Briefly, the mouse hind limbs were suspended by pulling the mouse’s tail so that the mouse only held the meter with its forelimbs. The mouse was gently pulled away, and the maximum force was recorded. Each mouse was tested 10 times, and each experiment was repeated 3 times.

Each mouse was placed in the open-field apparatus and recorded for 40 min. The movement and distance of mice were analyzed by Smart v 3.0 software. Each experiment was repeated 3 times.

### Statistical analysis

All data shown are mean ± SD. SPSS software (Statistical Product Service Solutions) was used for statistical analysis. Mann–Whitney U test was used for data with a non-normal distribution. Two-tailed Student’s *t* test was used for data with a normal distribution. One-way analysis of variance (ANOVA) followed by Bonferroni post hoc test was used for data with 3 groups. Statistical graphs are plotted by GraphPad Prism 8 (San Diego, CA, USA). Sample sizes and *P* values can be found in the figure legends. *P* values smaller than 0.05 were considered statistically significant.

## Data Availability

All data in the main text or the Supplementary Materials are available from the corresponding author (Y.C.). Raw data for transcriptome RNA sequencing are available in GSA-Human with project accession: PRJCA012457. Source data are provided with this paper.
