# Supplementary material for: Efficient Modulation of Exon Skipping via Antisense Circular RNAs
Source: Research (Wash D C). 2023 Jan 19;6:0045. doi: 10.34133/research.0045 (PMC10076032; doi:10.34133/research.0045)
Supplement: Supplementary 1 — Figs. S1 to S8 [file research.0045.f1.docx]

**Supplementary Figures**

**
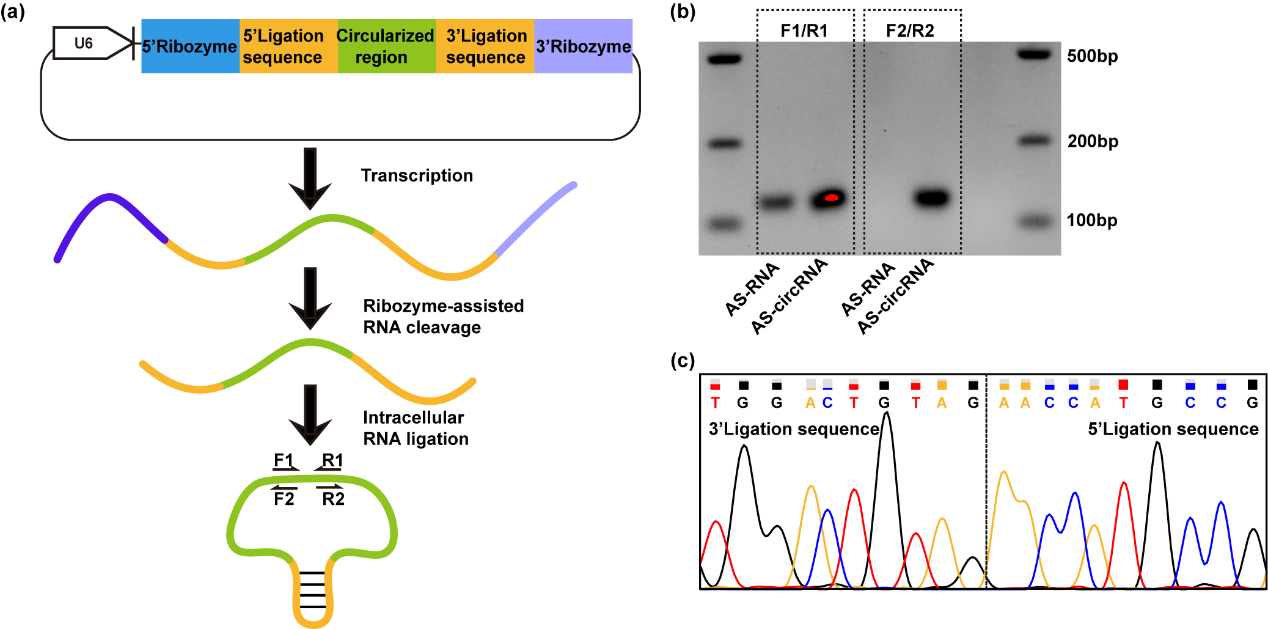
**

**Fig S1. Generation of AS-circRNAs in HEK293T cells.**

(a) Schematic diagram depicting the generation of genetically encoded circRNAs and the designed primer pairs. F1/R1 can amplify both linear and circular RNA. F2/R2 can amplify only circular RNA. (b) Confirmation of AS-RNA circularization using RT-PCR. (c) Sanger sequencing of RT-PCR products amplified by F2/R2 in the AS-circRNA group.

**
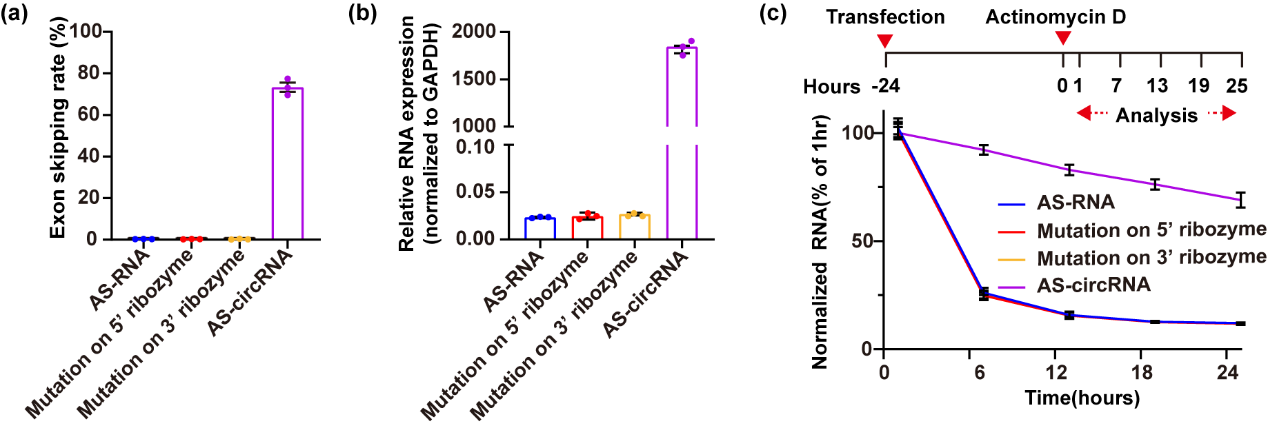
**

**Fig S2. Cyclization promotes the stabilization of antisense sequences.** (a) Exon skipping rates are mediated by different versions of RNA. Point mutations have been introduced separately to both ribozymes (Twister P3 U2A and Twister P1), necessary for cyclization. (b) The relative expression levels of AS-circRNAs with mutation in AS-circRNA precursor, normalized to GADPH. (c) Half-life analysis of different versions of RNA. Actinomycin D, a transcription inhibitor, was added 24 hours after transfection. Cells were collected 1, 7, 13, 19, and 25 hours after the addition of actinomycin D. Data are presented as mean±SD.


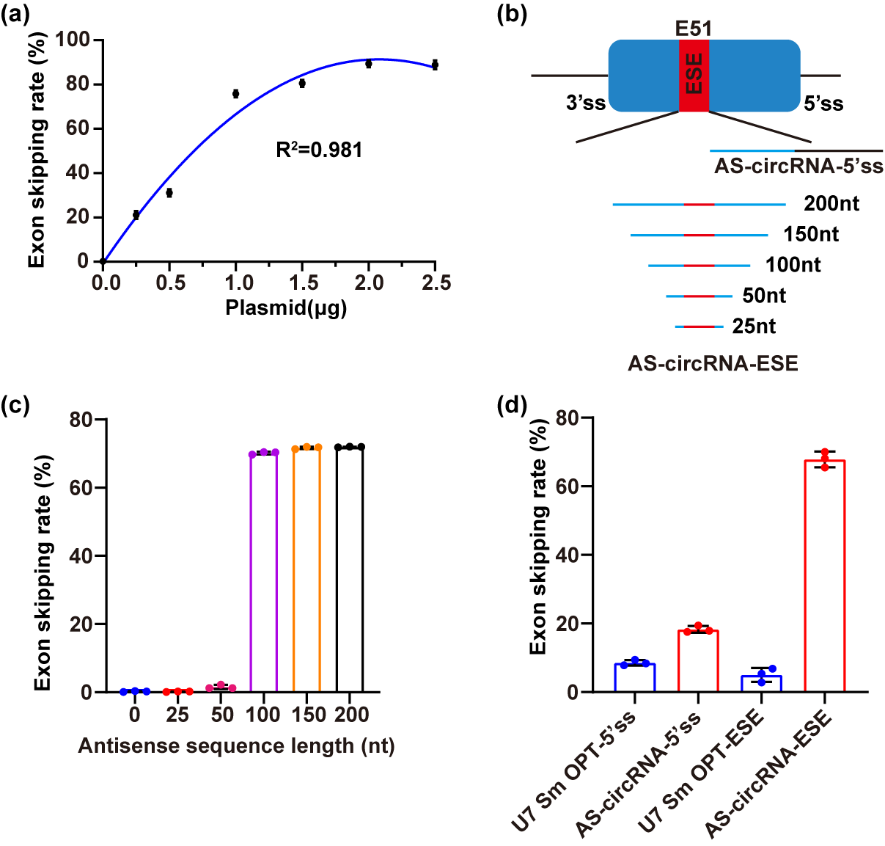


**Fig S3. The exon skipping rate depends on dose, antisense sequence length, and targeting elements.** (a) Exon skipping efficiency at different doses of the AS-circRNA plasmid, following the quadratic curve (y=-21.35X^2^+88.72X-0.82). (b) Schematic diagram of AS-circRNA with different lengths and targeting elements. AS-circRNA-5’ss targets the 5' splice site of E51, and AS-circRNA-ESE is designed to target the exonic splicing enhancer, ESE. The red region (+66+90) is the target of an ASO drug, and the antisense sequences of different lengths are shortened equally on both sides. (c) The exon skipping rate of AS-circRNAs with antisense sequences of different lengths. (d) The exon skipping rate of AS-circRNA and U7 Sm OPT in different targeting elements. Data are presented as mean±SD.

**
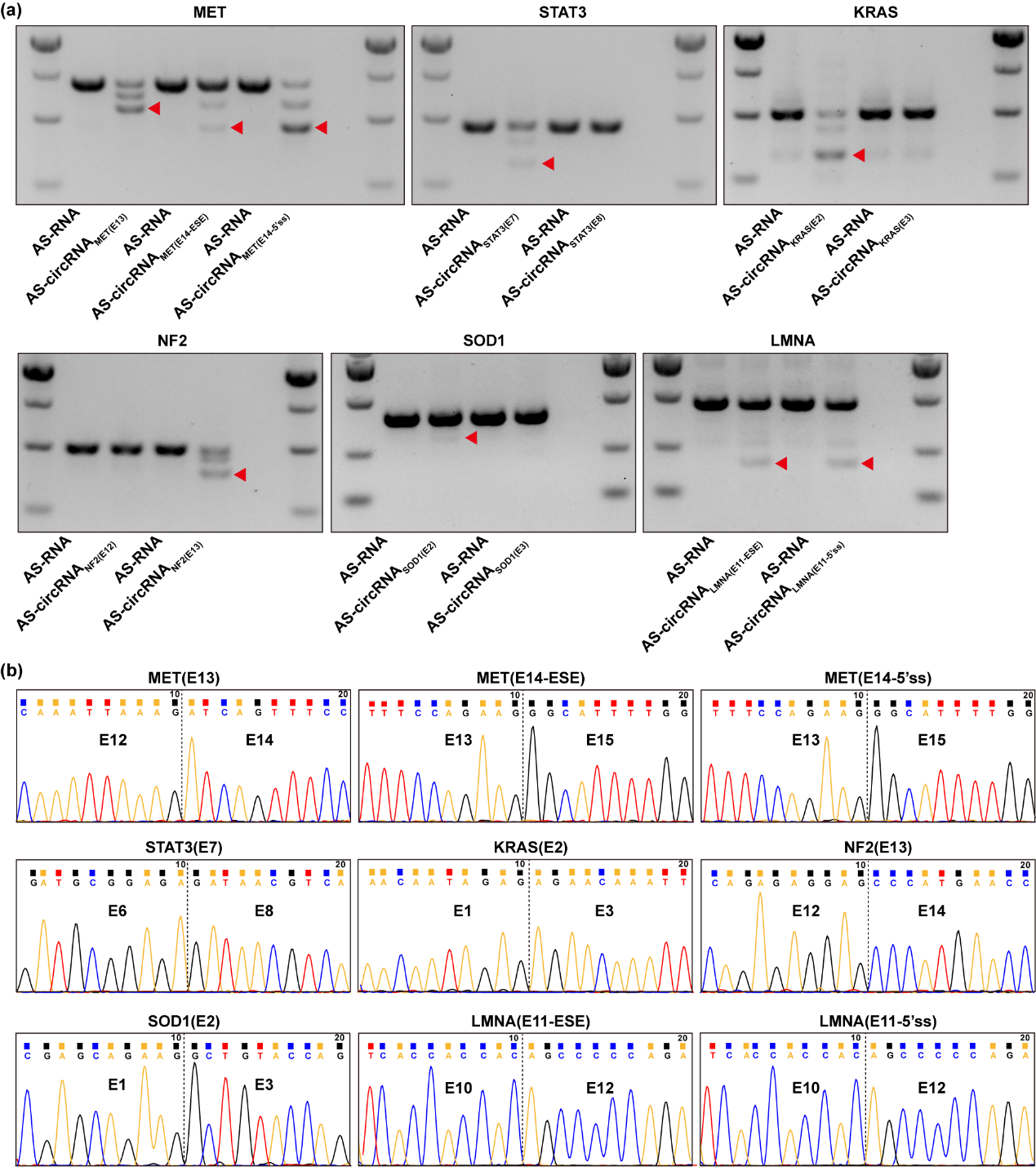
**

**Fig S4. As-circRNAs mediate exon skipping of endogenous transcripts in HEK293T cells.** (a) Effective exon skipping of endogenous transcripts mediated by nine AS-circRNAs. Red triangles indicate the skipped band. (b) Confirmation of the exon skipping by Sanger sequencing.


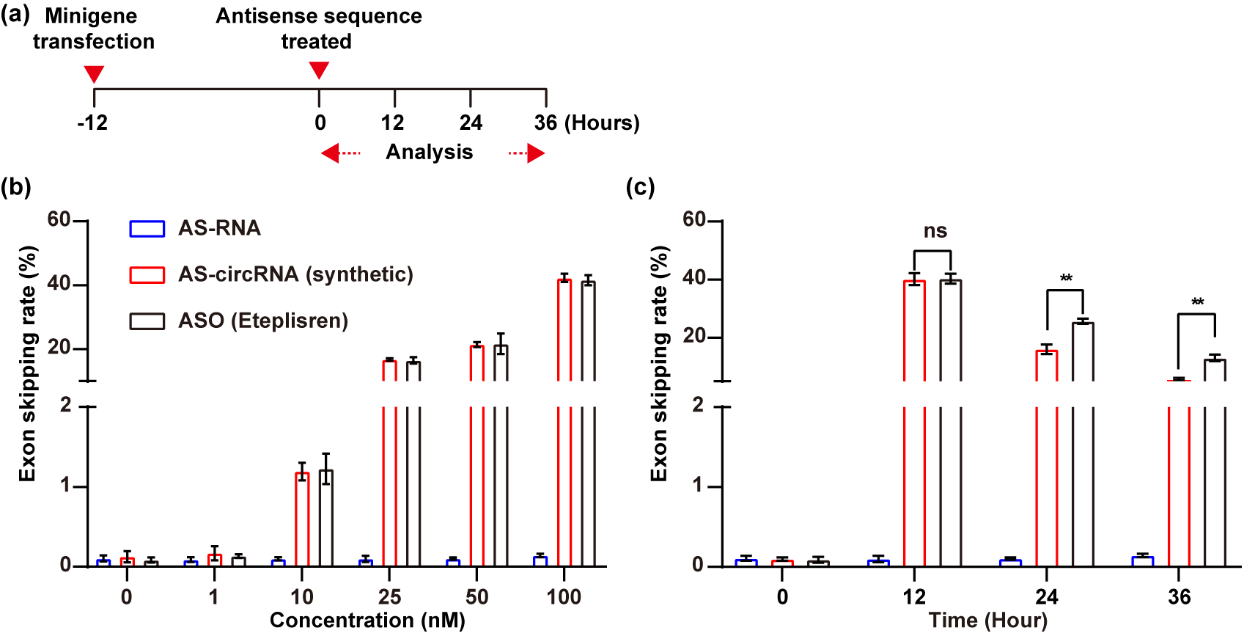


**Fig S5. AS-circRNAs synthesized *in vitro* mediate exons skipping in minigene transcript.** (a) Schematic diagram of experimental procedures. (b) AS-circRNAs synthesized *in vitro* showing comparable exon skipping efficiency to ASO (Eteplisren), an FDA-approved drug, at different concentrations after 12 hours treated. (c) Quantitative analysis of the exon skipping rate at different time points after transfection of AS-RNA, AS-cirRNA (synthetic), and ASO (Eteplisren) in HEK293T cells. Significance was determined by one-way ANOVA followed by Bonferroni post-hoc test. All data are presented as mean±SD. ***p* < 0.01, ns, *p*≥0.05.

**
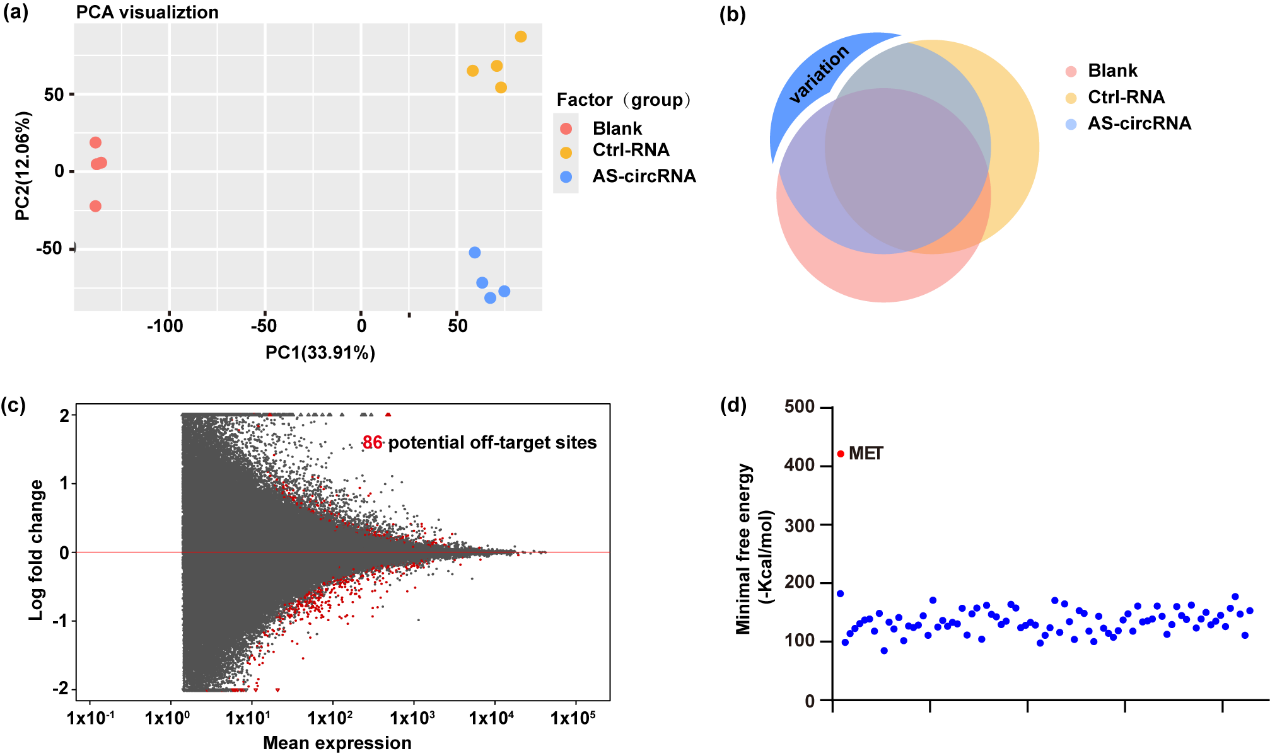
**

**Fig S6. AS-circRNAs show no detachable off-target effect at the transcriptomic scale.** (a) Principal component analysis (PCA) plot showing variation among 12 samples in three groups (AS-circRNA, Ctrl-RNA, and blank; n=4). (b) Venn diagram showing variation only in the AS-circRNA group compared to the other two groups. (c) Alternative splicing analysis showing all potential off-target sites, indicated by red dots (*p*<0.05). (d) Minimal free energy analysis showing that all of 86 off-target sites failed to form stable double-stranded structures with AS-circRNA, and thus are unlikely to be sequence-dependent off-targets. The red dots indicate binding to the target RNA.


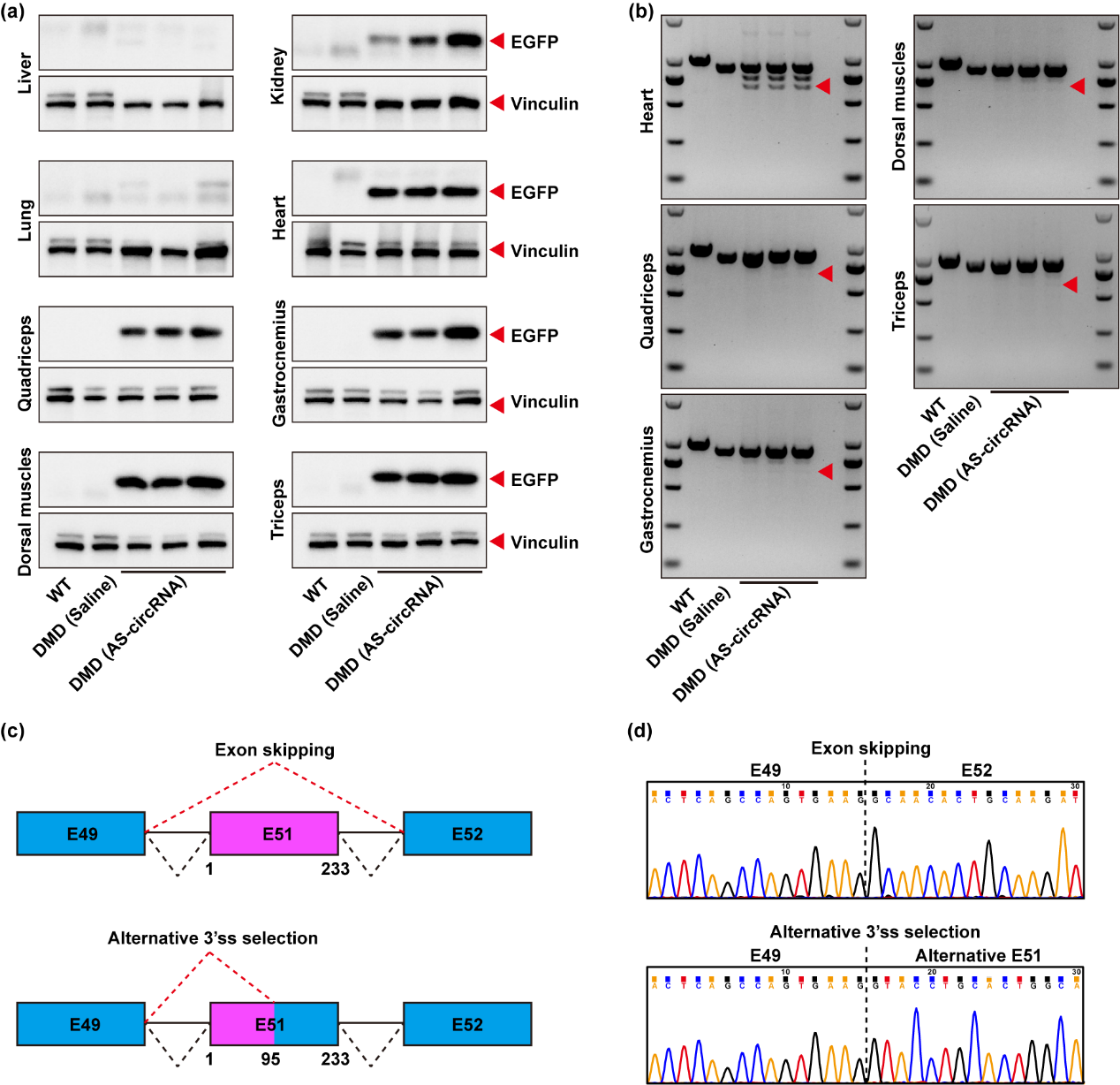


**Fig S7. MyoAAV delivered AS-circRNA mediates exon skipping in multiple muscle tissues.** (a) Western blot analysis showing EGFP expression in the kidney, lungs, heart, quadriceps, gastrocnemius, dorsal muscles, and triceps, but less in the liver, of DMD mice, four weeks after systemic injection of AS-circRNA packaged in scAAV. (b) Detection of exon 51-skipped mRNA by RT-PCR in heart, quadriceps, gastrocnemius, dorsal muscles, and triceps. (c) Schemic diagram of undesired bands caused by alternative 3’ss selection. (d) Sanger sequencing conforming the alternative 3’ss selection.

**
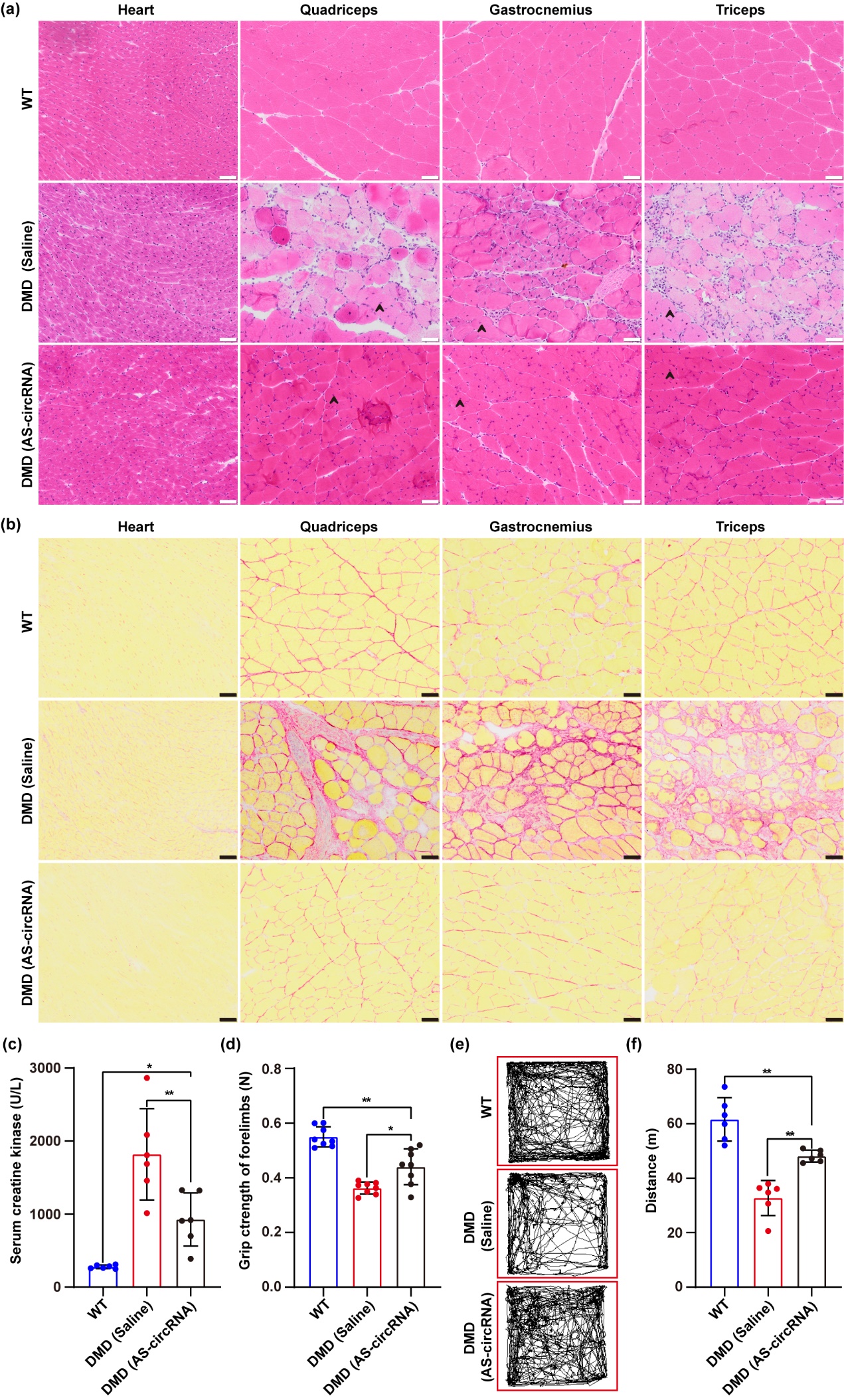
**

**Fig S8. AS-circRNAs improve the pathological phenotype and function of DMD mice.** (a) Hematoxylin-eosin staining of heart, quadriceps, gastrocnemius, and triceps in WT, DMD (Saline) and DMD (MyoAAV) mice. The black arrowhead indicates the muscle fibers with central nuclei. Scale bar, 50 µm. (b) Sirius red staining of heart, quadriceps, gastrocnemius, and triceps in WT, DMD (Saline) and DMD (MyoAAV) mice. Collagenous connective tissues were stained in red. Scale bar, 50 µm. (c) The creatine kinase (CK) level of WT, DMD (Saline) and DMD (MyoAAV) mouses. CK is a typical marker of muscle damage. n=6. (d) The forelimbs grip strength analysis of WT, DMD (Saline) and DMD (MyoAAV) mouses. n=8. (e and f) The analysis of movement trajectory and total travel distance within 20 minutes recording of WT, DMD (Saline) and DMD (MyoAAV) mice. n=6. All data are presented as mean±SD. One-way ANOVA followed by Bonferroni post-hoc test was used for significant analysis. **p* < 0.05, ***p* < 0.01.
